# Depth sets the recovery clock: evidence for a time-at-pressure effect in sea lions

**DOI:** 10.1098/rspb.2026.1690

**Published:** 2026-10-28

**Authors:** Jonas F. L. Schwarz, Daniel P. Costa, Oliver Krüger, Nathan Angelakis, Alastair Baylis, Barbara L. Chilvers, Carsten F. Dormann, Shannon L. Fowler, Simon D. Goldsworthy, Robert Harcourt, Joseph I. Hoffman, Luis A. Huckstadt, Jana Jeglinski, Elizabeth McHuron, Clive R. McMahon, Sharon Melin, Diego Páez-Rosas, Svenja Stoehr, Fritz Trillmich, Stella Villegas-Amtmann, Rachel R. Holser

**Affiliations:** ^1^ Institute of Marine Sciences, University of California Santa Cruz, Santa Cruz, CA, USA; ^2^ Department of Ecology and Evolutionary Biology, University of California Santa Cruz, Santa Cruz, CA, USA; ^3^ Biometry and Environmental System Analysis, University of Freiburg, Freiburg, BW, Germany; ^4^ Joint Institute for Individualisation in a Changing Environment (JICE), Bielefeld University, Bielefeld, Germany; ^5^ Department of Animal Behaviour, Bielefeld University, Bielefeld, Germany; ^6^ Department of Evolutionary Population Genetics, Bielefeld University, Bielefeld, Germany; ^7^ Center for Biotechnology (CeBiTec), Faculty of Biology, Bielefeld University, Bielefeld, Germany; ^8^ Ecology and Evolutionary Biology, School of Biological Sciences, The University of Adelaide, Adelaide, Australia; ^9^ South Australian Research and Development Institute (Aquatic Sciences), West Beach, Australia; ^10^ South Atlantic Environmental Research Institute, Stanley, Falkland Islands; ^11^ Department of Conservation, Marine Species and Threats Team, Wellington, New Zealand; ^12^ WILDBASE, Institute of Veterinary, Animal and Biomedical Science, Massey University, Palmerston North, New Zealand; ^13^ Department of Life Sciences, University of Roehampton, London, UK; ^14^ Marine Predator Research Group, School of Natural Sciences, Macquarie University, Sydney, Australia; ^15^ British Antarctic Survey, Cambridge, UK; ^16^ Centre for Ecology and Conservation, University of Exeter, Penryn, UK; ^17^ Sharjah Marine Science Research Centre, University of Khorfakkan, Sharjah, United Arab Emirates; ^18^ Department of Ecoscience, Aarhus University, Aarhus, Denmark; ^19^ School of Biodiversity, One Health and Veterinary Medicine, University of Glasgow, Glasgow, UK; ^20^ Cooperative Institute for Climate, Ocean, and Ecosystem Studies, University of Washington, Seattle, WA, USA; ^21^ IMOS Animal Tagging, Sydney Institute of Marine Science, Mosman, Australia; ^22^ National Marine Mammal Laboratory, Alaska Fisheries Science Center, Seattle, WA, USA; ^23^ Galapagos Science Center, Universidad San Francisco de Quito, Islas Galápagos, Ecuador; ^24^ Fundación Conservando Galápagos, Galapagos Conservancy, Puerto Baquerizo Moreno, Ecuador; ^25^ Dirección del Parque Nacional Galápagos, Unidad Técnica Operativa San Cristóbal, Puerto Ayora, Ecuador

**Keywords:** post-dive duration, respiratory physiology, marine mammals, pressure linked inert-gas loading

## Abstract

All air-breathing marine vertebrates face the same fundamental trade-off: time spent foraging underwater must be offset by surface recovery between dives. For decades, the dominant view in diving physiology has been that post-dive duration (PDD) reflects the repayment of oxygen debt and clearance of carbon dioxide and lactate. Using more than 800 000 dives from five sea lion species across 18 colonies spanning tropical to subpolar waters, we quantified how PDD scales with dive depth and duration and evaluated whether this pattern is consistent with metabolic recovery interpretations or instead points to depth-linked constraints. We found that PDD increases primarily and linearly with depth, while dive duration contributes to PDD only at greater depths, a relationship conserved across species despite pronounced differences in body mass, dive physiology and foraging ecology. Our findings conflict with predictions of metabolic recovery interpretations and point to a pressure-linked mechanism in which time at depth and resulting inert-gas loading shape surface recovery. By highlighting pressure-linked inert-gas management as a central candidate mechanism for surface interval regulation in inhalation divers, our study revises a central assumption in diving physiology, with broad consequences for understanding foraging efficiency, habitat accessibility and vulnerability to environmental change in air-breathing marine vertebrates.

## 1. Introduction

The importance of post-dive duration (PDD)—the surface interval between consecutive dives—is well understood by human divers, who follow decompression tables that scale recovery time with both dive depth and duration to manage inert nitrogen (N_2_) and avoid decompression sickness [1]. Highly specialized air-breathing marine vertebrates such as marine mammals exhibit pronounced surface intervals [2], but with adaptations such as depth-induced pulmonary/alveolar collapse that are assumed to limit gas exchange at depth [3,4], PDD in these species is typically interpreted as the time needed to restore oxygen stores and clear carbon dioxide and lactate [5–8]. Some authors, however, contend that these adaptations are insufficient to prevent complete nitrogen accumulation, advocating a shift in perspective from minimizing nitrogen uptake alone to considering the explicit management of nitrogen load across the dive cycle, a view that gained renewed attention after gas emboli pathology was reported in association with mass strandings of deep-diving beaked whales (e.g. [9]). Comparative evidence further indicates that PDD in marine mammals and diving birds scales with both dive duration and depth [10], prompting renewed scrutiny of the mechanisms underlying surface recovery time.

Understanding what governs PDD has implications beyond refining physiological limits and gas exchange models [11]. Because recovery intervals constrain time spent underwater, they directly influence foraging efficiency [12] and, in turn, affect energy budgets, exposure to predators, reproductive performance and population dynamics [13,14]. If depth-linked time-at-pressure processes are major drivers of recovery, then strict metabolic-centric models may misallocate energetic costs, biasing estimates of foraging efficiency, habitat use and sensitivity to disturbance [8,10,15–18]. Clarifying whether PDD is governed chiefly by metabolic recovery, depth-related processes, or their interaction is central to building a general predictive theory of diving physiology [11]. To date, most empirical studies of dive physiology have focused on single species and environments (e.g. [8,19]). Developing a broadly applicable principle requires comparative designs integrating both intra- and interspecific variation in body sizes, dive physiologies and environmental contexts to make inferences that are robust, integrative and generalizable across taxa and ecosystems.

We use a global biologging dataset comprising more than 800 000 dives from 374 individuals across five sea lion species from 18 colonies worldwide (figure 1) [22–33] to quantify the relationship between dive depth, dive duration and PDD, and to evaluate how well the emergent pattern we observed aligns with the currently accepted interpretations of surface recovery. Sea lions provide an exceptional model system for addressing this question because they span a wide range of body masses [20], exhibit contrasting dive physiologies [2,19,21,34,35] and employ diverse diving strategies, from epi- and mesopelagic to benthic foraging [27,28,31–33]. They also occupy diverse environments ranging from tropical to subpolar waters (figure 1). Importantly, otariids, like many marine mammals and diving birds, dive on inhalation and carry substantial pulmonary air stores [4,36,37], making them particularly well-suited to evaluating pressure-linked constraints on recovery. Together, this allows us to establish how PDD scales with depth and duration across broad physiological, behavioural and environmental conditions, and to then evaluate how well metabolic recovery expectations versus alternative, depth-dependent explanations account for the observed pattern.

**Figure 1.**
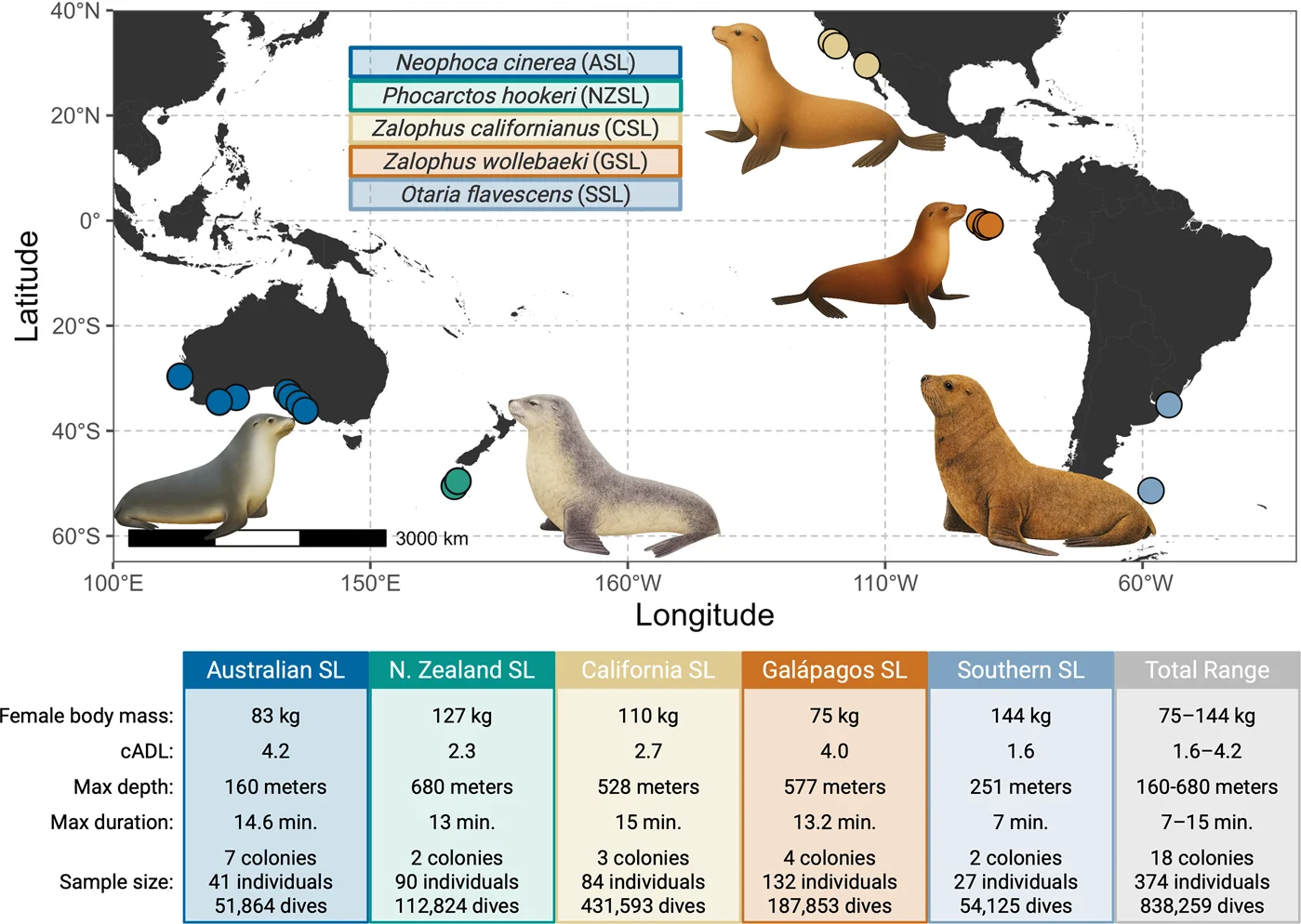
Overview of our dataset, including geographical distributions and physiological parameters of the five sea lion species. Shown are colonies sampled and mean female body mass [20], calculated aerobic dive limit in minutes (cADL) [21] and maximum dive depth and duration, calculated from the dataset used in this study after excluding the top 1% of the deepest and longest dives to remove potential extreme outliers. Created in BioRender, https://BioRender.com/tzdgh7u.

## 2. Results

Maximum dive depth is the primary driver of PDD in our dataset (figure 2). At mean maximum depth and duration, the intercept is 111 s (approx. 1.9 min, see supplementary material, table S1, for the full linear mixed-effect model output) and each additional 10 m of depth increases PDD by 8.74 s (SE 0.73) when evaluated at a representative dive duration of 7 min. By contrast, the main effect of duration at mean depth is weaker and slightly negative (−0.50 s per +10 s; SE 0.02). This dominance of depth is also apparent in the raw dive data, where PDD gradients align primarily with the depth axis in the heatmap (figure 2A). Together, these results indicate that dive depth is the principal determinant of PDD, not dive duration.

**Figure 2.**
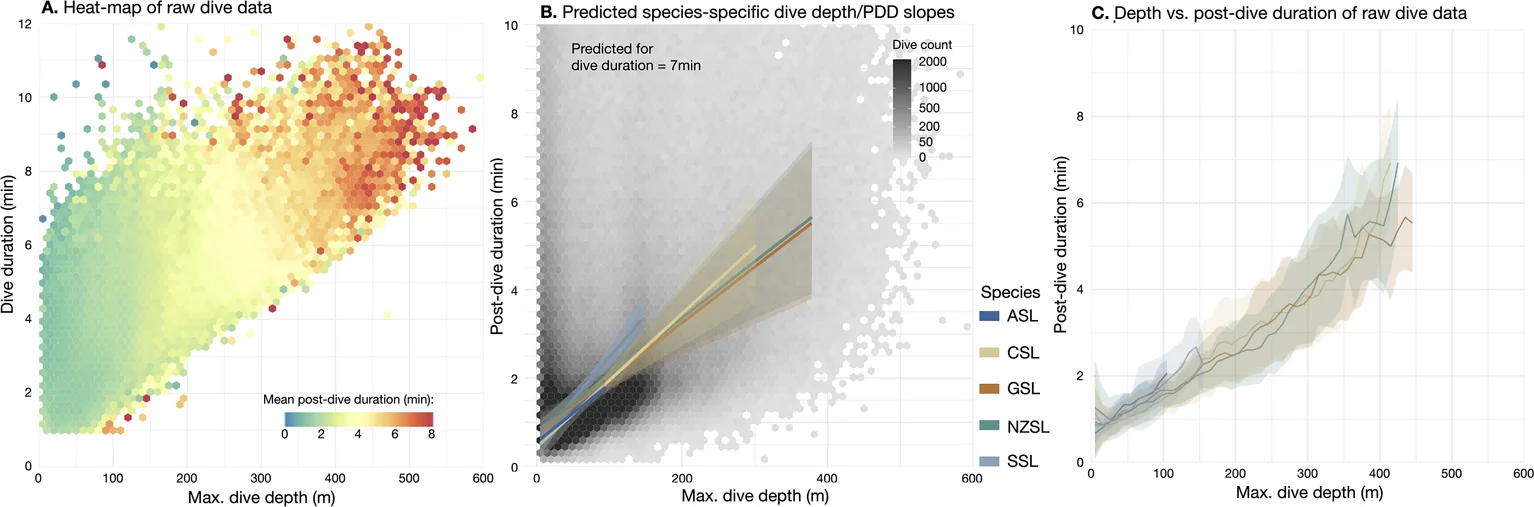
Depth dependence of post-dive duration (PDD). (A) Hexbin heatmap of the raw dive data showing the mean PDD (fill) across the depth–duration plane (correlation between depth and duration: *r* = 0.826). (B) Species-specific model predictions (mean ± 95% CI) for the relationship between PDD (minutes) and maximum dive depth (metres) derived from the random-effects structure of the linear mixed-effects model and conditioned on a representative 7-min dive; the grey hex background depicts the density of the data based on all observed dives, irrespective of dive duration, using a square-root scale. (C) Smoothed relationships between PDD (minutes) and maximum dive depth (metre) for the raw dive data. Dive depth was binned in 10-m intervals and bins with less than 100 dives were excluded. Species medians with respective IQR ribbons are shown.

The slope of the relationship between dive depth and PDD is similar across all five sea lion species, indicating consistent effects of dive depth on recovery time (figure 2B). Model predictions conditioned on a representative 7-min dive show tightly overlapping slopes across species, with modest species-level random-effect variances (SD = 9.9 s for intercepts; depth-slope SD = 1.55 s per 10 m) and individual-level variation exceeding species-level variation (intercept SD = 23.3 s; depth-slope SD = 3.19 s per 10 m). This pattern is also evident in the raw data (figure 2C). Conditioning the model predictions for visualization on 7-min keeps the results within a dense, biologically plausible region of the depth–duration space (figure 1), while avoiding unrealistic combinations of short durations with extreme depths. The density background in figure 2B, however, shows all observed dives with different dive durations, so its highest density regions are not expected to align perfectly with predictions at a fixed dive duration.

Dive duration influences PDD primarily through its interaction with depth. The interaction between depth and duration is strongly positive, with PDD increasing by 0.21 s for each combination of a 10 m depth and 10 s duration increase, indicating that, while the main effect of duration is slightly negative at mean depth, its net effect on PDD is negligible for shallow dives but increases markedly as depth increases. This is illustrated by the model response surface (figure 3A), where PDD increases with increasing duration at greater depths. Similarly, raw data relationships show almost no effect of duration on PDD at 50–70 m depth (figure 3B), a modest effect at 150–170 m (figure 3C) and a pronounced positive relationship at 250–270 m (figure 3D). This suggests that, as dives get deeper, the combined effect of duration and depth significantly prolongs surface recovery intervals.

**Figure 3.**
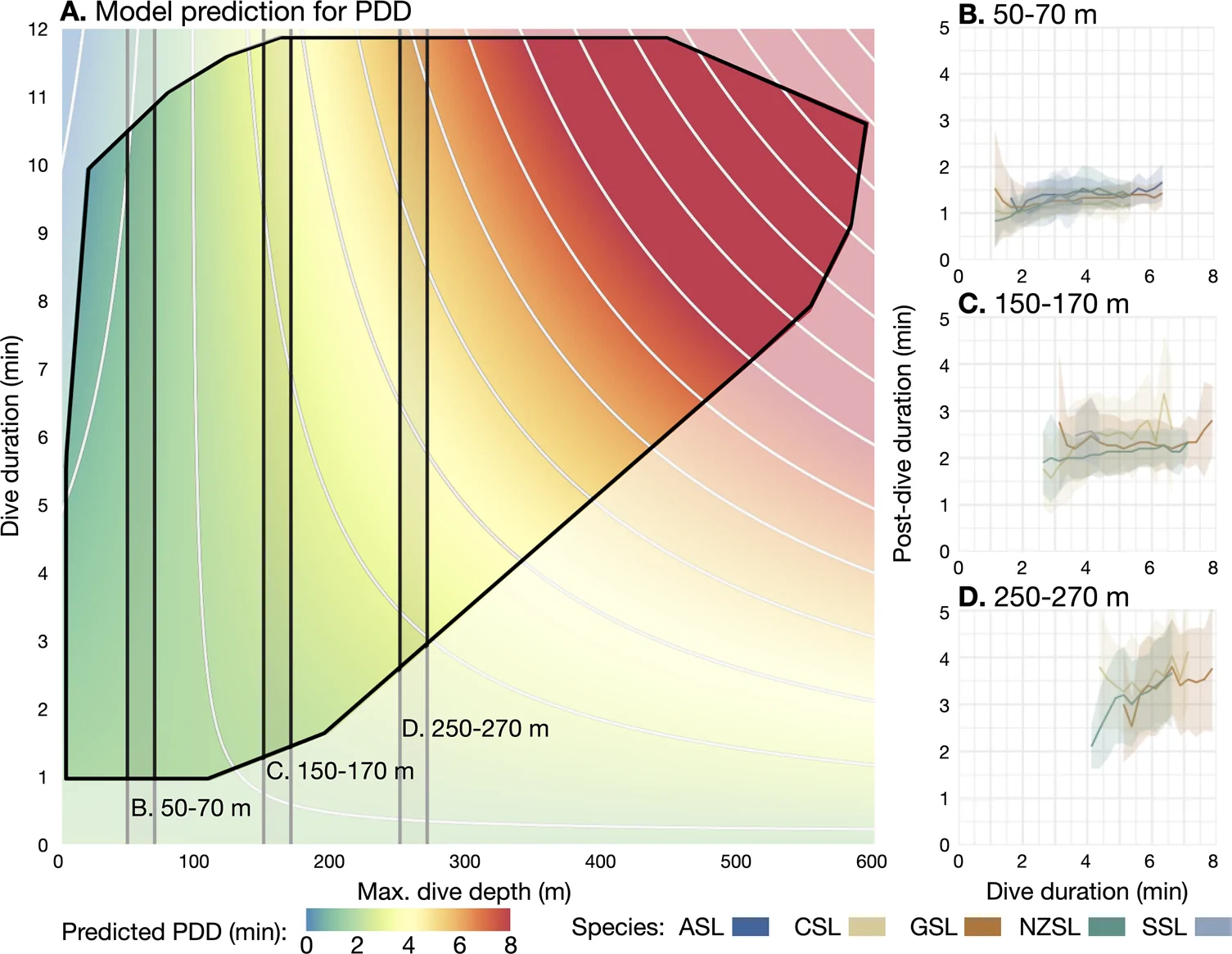
Depth-dependent effect of dive duration on PDD. (A) Response surface from the mixed-effects model (PDD in minutes; colour scale and white isopleths), plotted over the convex hull of observed depth–duration combinations (black polygon). Vertical black lines mark the three depth bins corresponding to panels B–D. (B–D) Smoothed relationships between dive duration and PDD (minutes) of raw dive data of three depth bins: 50–70 metres (B), 150–170 metres (C) and 250–270 metres (D). Dive duration was binned at 0.25 min, dropping bins with less than 25 dives. Species medians with their interquartile ribbons are shown.

## 3. Discussion

In the field of diving physiology, PDD is typically viewed as the time needed to replenish oxygen stores and clear byproducts of aerobic and anaerobic metabolism (carbon dioxide and lactate) [5–8]. This metabolic recovery view predicts that PDD should increase with dive duration, especially when exceeding the aerobic dive limit (ADL) [5,8,16]. As dive energetics govern the rate at which oxygen is used [10], oxygen consumption and carbon dioxide production are also affected by body size, metabolic rate and diving behaviour [2,19,34,35]. Species that differ in these traits are thus expected to show different scaling ratios of recovery time with dive duration, with additional variation in oxygen stores and ADLs further reinforcing this expectation [8,21]. However, our findings across five sea lion species challenge this view and highlight the role of depth (i.e. pressure) in determining recovery time at the surface.

### (a) Limitations of the metabolic recovery view

Any metabolic recovery explanation of PDD must account for three features of the dataset simultaneously: PDD increases nearly linearly with dive depth; the depth–PDD scaling is strikingly similar across five sea lion species despite large differences in body mass, oxygen stores, metabolic rates and foraging strategies; and dive duration influences PDD primarily through a depth × duration interaction rather than as a broadly positive main effect. While each feature can be rationalized in isolation, reconciling all three within a metabolic recovery framework is difficult.

First, if PDD chiefly reflects metabolic recovery after a dive, whether framed in terms of oxygen replenishment, carbon dioxide clearance/acid–base balance and/or lactate processing, then any effect of depth on PDD should arise largely through depth-related changes in locomotor effort and energetic cost [10]. Yet the relationship between depth and energetic cost is not straightforward and varies across species and studies (e.g. [8,19]), with some studies reporting even lower costs of deeper dives due to buoyancy changes that permit prolonged glides [8]. Furthermore, energy requirements of deeper dives are expected to scale nonlinearly with depth [35,38], including an energetic optimum near roughly twice the depth of neutral buoyancy [19,39]. Instead, and contrary to these predictions, we find a clear linear increase of PDD with depth, with no sharp increase in PDD for the deepest dives, as would be expected if lactate clearance once ADL was exceeded was a major contributor to PDD [5,8,16]. This linearity is difficult to reconcile with metabolic recovery interpretations that typically imply non-linear depth-cost relationships. However, linearity by itself does not rule out a metabolic recovery interpretation, because body mass, physiology and dive behaviour could still modulate recovery; the key point is whether this scaling changes across different body masses, physiologies and dive behaviours.

Second, if the depth–PDD relationship primarily reflects metabolic recovery, it should rescale across the five sea lion species. This expectation follows because body mass, dive physiology (including oxygen stores and metabolic rates) and foraging/locomotor strategies, and thus energetic costs, differ markedly among them [2,19,34,35,40]. Instead, PDD increases with depth at remarkably similar rates across all five species, with most of the remaining variation expressed at the individual level rather than as systematic species differences in depth scaling. This near-parallel scaling suggests that the dominant process linking depth to recovery is not strongly influenced by species-specific differences in body size, physiology or dive behaviour and thus energetics.

Finally, metabolic recovery interpretations predict that longer dives should generally require longer surface intervals because oxygen depletion and carbon dioxide accumulation increase with breath-hold duration, and recovery processes (reloading oxygen stores, clearing carbon dioxide/acid load and, when relevant, processing lactate) take time [5–8]. Under this view, dive duration should exert a broadly positive effect on PDD across depths, potentially steepening once dives exceed ADL [5,8,16]. Instead, dive duration has little influence on PDD for shallow dives, yet matters increasingly for recovery as depth increases. This depth-dependent effect of duration is also reflected in the gradual steepening of the raw depth–PDD smooths (figure 2C), because deeper dives are generally longer and therefore involve more time spent under pressure. Duration thus becomes consequential primarily under high-pressure conditions, indicating that PDD is not a simple function of breath-hold time but depends on depth-specific constraints.

### (b) A pressure-linked mechanism for PDD

The combined structure of the depth–duration–PDD relationship across all five species is difficult to reconcile with metabolic recovery interpretations alone, and is more consistent with pressure-linked constraints. The linear increase of PDD with depth and the depth-dependent effect of duration are more consistent with constraints imposed by hydrostatic pressure [41]. Sea lions, like many marine mammals and diving birds, dive on inhalation [4,36,37] and therefore carry a significant amount of air in their lungs. As they dive deeper, lung compression elevates alveolar gas pressures, increasing the gradient for gas uptake, such as nitrogen, into their blood and tissues [11]. As nitrogen is not metabolized, it accumulates with time at depth [3,42]. Longer PDDs may thus allow partial off-loading of accumulated nitrogen, particularly from faster-equilibrating tissues, thereby slowing cumulative nitrogen loading across repeated dives and reducing the risk of supersaturation and bubble formation [42].

Our results bear clear hallmarks of a time-at-pressure process. First, PDD increases linearly with depth, mirroring the rise in hydrostatic pressure and the associated potential for nitrogen uptake [11], with longer PDD intervals potentially slowing cumulative nitrogen loading [3,42]. Second, the slope of the depth–PDD relationship is largely conserved across the five sea lion species, consistent with a biophysical process that is comparatively less sensitive to species differences in oxygen stores or metabolic rates [11]. Third, dive duration becomes important mainly at greater depths, as would be expected if nitrogen load scales with time spent under pressure [11]. Together, these patterns are most consistent with a time-at-pressure mechanism. Although individual parts of this pattern could potentially be explained by alternative physiological or behavioural processes, any alternative explanation must account for the combined structure of the results. Nitrogen management provides one coherent candidate mechanism for this combined pattern, as nitrogen uptake and off-gassing are expected to depend on both pressure and time at pressure [43].

Oxygen and carbon dioxide dynamics may also contribute to pressure-linked recovery, as both gases are affected by pressure, while CO_2_ has independently been shown to influence post-dive ventilatory behaviour [44]. However, unlike nitrogen, O_2_ and CO_2_ are metabolically active gases: O_2_ is consumed and CO_2_ produced during a dive. While nitrogen dynamics are also expected to vary among species due to differences in body mass, diving lung volume, pulmonary shunting, body composition, ascent behaviour and repeated-dive history [42], O_2_/CO_2_ dynamics should additionally reflect species- and behaviour-specific differences in metabolic rate, activity, ventilatory regulation, buffering capacity, dive response and foraging strategy [10]. A recovery mechanism dominated by O_2_/CO_2_ dynamics would therefore be expected to show stronger species- and behaviour-specific scaling. Similar CO_2_ tolerance or convergent ventilatory regulation across species could contribute to the observed similarity, but would not explain why duration becomes consequential mainly at greater depths. Thus, while CO_2_ can influence surface recovery, the observed depth-dependent effect of duration suggests that pressure-linked dynamics become increasingly important with depth. Although the physiological pathway linking nitrogen accumulation to behavioural regulation is currently unknown, the observed depth- and duration-dependent adjustment of PDD suggests that sea lions may respond indirectly to physiological consequences of nitrogen loading or to correlated pressure-linked cues.

This interpretation of the observed patterns relies on the possibility that pulmonary gas exchange can continue, at least partially, during deep dives. If exchange effectively ceased beyond the assumed lung-collapse depth, the depth–PDD relationship should plateau or inflect once dives exceed that threshold (225 ± 25 m in California sea lions; [4]). Instead, we observe a continuous, linear increase in PDD across the observed depth range. This challenges the strict interpretation that lung collapse at depth fully halts further gas exchange [3,43,45]. While pressure-chamber work suggested strong pulmonary shunting at depth [46], more recent studies in free-diving California sea lions indicate that lung collapse is not a fixed threshold and may not be equivalent to complete pulmonary shunt [4,38,47]. Physiological measurements of high arterial and central venous oxygen levels at depths beyond predicted collapse have been interpreted as inconsistent with the complete cessation of pulmonary exchange, even when accounting for A–V shunting and partial arterialization of central venous blood [4,38,47], and theoretical models incorporating continued pulmonary gas exchange after partial lung collapse similarly predict greater nitrogen loading with increasing depth and time-at-pressure (e.g. [43]). Together, these lines of evidence indicate that some gas exchange likely continues beyond the presumed collapse depth, permitting ongoing nitrogen uptake, while lung volume, collapse depth, pulmonary shunting, perfusion and repeated-dive history likely modulate the magnitude and timing of that uptake.

Direct physiological evidence for these dynamics currently comes primarily from California sea lions [4,38,47], and comparable blood–gas measurements are not available for all species included here. However, all five species are otariids and share the broad respiratory anatomy and inhalation-diving strategy that make pressure-linked pulmonary gas exchange relevant [4,36,37]. While species-specific differences in collapse depth or pulmonary shunting may modify the magnitude and timing of nitrogen uptake, they do not preclude a shared pressure-linked mechanism contributing to the conserved depth–PDD pattern observed across sea lions.

### (c) Taxonomic scope beyond sea lions

Dive depth, independent of dive duration, has been found to affect PDD across marine mammals and diving birds [10], and the possibility of time-at-pressure effects as a contributing factor to PDD should be considered beyond sea lions. However, the expression of gas management likely differs by taxon due to morphological differences and behavioural adaptations. While otariids typically dive on inhalation [4], as do many cetaceans [37] and diving birds [36], many phocids exhale before diving, relying proportionally less on lung oxygen [3,48], making complete collapse/no-gas-exchange at depth more plausible for some phocids [49]. A recent study in northern elephant seals (*Mirounga angustirostris*), an exhalation-diving phocid, aligns with this expectation, showing that PDD was insensitive to dive depth [50]. Another factor is body composition, because long time-constant tissues such as fat equilibrate more slowly to nitrogen loads than other tissues [37]. While otariids have relatively little blubber compared to other pinnipeds [51], species with more blubber, like phocids or cetaceans, may have a higher capacity to store larger transient nitrogen loads and therefore may rely more on sequence-level decompression behaviour than on extended PDD [37,52], potentially allowing them to move decompression times to the end of dive bouts. In addition, surface time is not explicitly necessary for decompression. Shallow ‘decompression-style’ dives between deep dives would also allow nitrogen off-loading, which has been discussed for some species of beaked whales and king penguins [37,52]. Thus, while the basic pressure–time logic can be generalized, mechanistic pathways may differ among taxa.

### (d) Implications

Our findings challenge the long-held assumption that PDD predominantly reflects metabolic recovery, an interpretation that has often been emphasized despite longstanding recognition that inert-gas constraints may also matter [3,9]. Without modelling gas dynamics explicitly, we use a large comparative dataset to identify a simple, general scaling relationship between depth, duration and recovery time that places strong constraints on metabolic explanations, including activity-mediated oxygen and carbon dioxide frameworks. In this context, nitrogen management, potentially interacting with pressure-linked O_2_ and CO_2_ dynamics, emerges as a leading candidate mechanism shaping surface recovery time in sea lions, a clade of inhalation divers [4]. However, because nitrogen uptake and tissue nitrogen load were not measured directly, we cannot exclude that other, currently unresolved pressure-linked mechanisms could generate a similar depth–duration–PDD pattern. Because dive depth, independent of dive duration, has been found to affect PDD across marine mammals and diving birds (a pattern previously attributed to increased oxygen consumption) [10], pressure-linked inert-gas loading should be broadly considered as a potential contributor to PDD. By reframing PDD as recovery from a time-at-pressure process, nitrogen handling is placed as a promising potential mechanism alongside oxygen, carbon dioxide and lactate management, modifying a central assumption that has guided diving physiology for decades.

The implications of our findings extend beyond dive physiology. Depth-linked increases in PDD directly reduce foraging efficiency, sharpening the trade-off between the benefits of accessing deep foraging habitats and incurring longer surface recovery times [8,12]. These insights clarify the drivers of cross-species and colony differences in realized niche use and provide a framework for understanding and predicting the ability of marine mammals to respond to environmental change [40]. Ultimately, by shifting the focus to physical constraints, our study provides new insights into how air-breathing inhalation divers exploit a diverse ocean.

## 4. Material and methods

### (a) Animal handling and ethics

We synthesized biologging datasets collected by multiple research groups on free-ranging sea lions under permits and approvals granted by national and institutional authorities, including (but not limited to) the US National Marine Fisheries Service, the Argentine National Council for Scientific and Technological Research, the South Australian Department for Environment and Water, the Falkland Islands Government, the New Zealand Department of Conservation and the Ecuadorian Ministry of Environment. Original studies [22–33] followed established best practices to minimize disturbance. Devices were attached with temporary, non-invasive methods and, where required, immobilizations were performed by trained personnel under veterinary oversight. No new animal handling was conducted for this synthesis. All procedures and permits are detailed in the original publications.

### (b) Study species, sites and deployments

Data span five sea lion species monitored at 18 colonies worldwide (Galápagos sea lion colonies: Caamaño, Isla Floreana, Isla Fernandina, Malecón San Cristóbal; California sea lion colonies: Isla Granito, San Miguel Island, San Nicolas Island; New Zealand sea lion colonies: Dundas Island, Enderby Island; Southern sea lion colonies: Falkland, Isla de Lobos; Australian sea lion colonies: Dangerous Reef, Lilliput Island, Olive Island, Seal Bay, Six Mile Island, Fisherman Islands, Kimberley Island) between 2000 and 2023. Across all sources, we analysed dive data from 374 lactating females. Archival time-depth recorders (TDRs) were deployed for a median of approximately 3 weeks (range approx. 2–104 days) and configured to provide depth at 4-s resolution. Higher-frequency records (when present) were down-sampled to 4 s to standardize sampling frequency across datasets.

### (c) Dive identification and basic metrics

Depth time series were processed in R with *diveMove* [53]. We applied a variable zero-offset correction (ZOC) tuned per individual to reduce sensor drift, then identified dives using a minimum depth threshold of 5 m and a minimum duration of 60 s, to exclude shallow and short surface dives, resulting in 801 086 dives. For each dive, we extracted maximum depth, dive duration and post-dive duration (PDD), defined as the elapsed time from the end of a dive to the start of the next dive [54]. PDDs longer than 30 min, found for example for the last dive of a dive bout, were excluded, as they exceeded by far the usual observed recovery time and are more likely connected to other behavioural states.

### (d) Statistical modelling

We fitted a linear mixed-effects model, where *y* denotes PDD in seconds, *d* maximum depth (m) and *t* dive duration (s), with standardized predictors (mean 0, SD 1):

y∼z(d)∗z(t)+(1+z(d)||species)+(1+z(d)||individual)

where individual is a unique animal identifier. Random-effects terms were specified with uncorrelated intercepts and depth-slope components per grouping factor. Model-frame scaling attributes (means and SDs) were retained for prediction. The complete model output including effect sizes is reported in supplementary material, table S1. Because model residuals exhibited substantial heterogeneity, we assessed robustness to alternative transformations of the response (log, square-root, rank), non-linear predictors (2nd order polynomials) and alternative random-effects structures (with/without random intercepts or slopes; species as fixed effect). Qualitative conclusions (sign and relative magnitude of main effects and the interaction) were unchanged across these specifications.

### (e) Software

Analyses were conducted in R (version 4.5.0; 2025-04-11), using the package lme4 [55].

## Supplementary Material



## Data Availability

All data and code necessary to reproduce the analyses presented in this study are available from [54]. Supplementary material is available online [56].
