# Supplementary material for: Depth sets the recovery clock: evidence for a time-at-pressure effect in sea lions
Source: Proc Biol Sci. 2026 Oct 28;293(2081):20261690. doi: 10.1098/rspb.2026.1690 (PMC13725537; doi:10.1098/rspb.2026.1690)
Supplement: Supplementary file 1 [file rspb.2026.1690.zip › rspb.2026.1690/rspb20261690_si_001.pdf]

Supporting Information for

Depth sets the recovery clock: evidence for a time-at-pressure effect in sea lions.

Proceedings of the Royal Society B: DOI: 10.1098/rspb

Jonas F.L. Schwarz, Daniel P. Costa, Oliver Krüger, Nathan Angelakis, Alastair Baylis, B. Louise Chilvers, Carsten F. Dormann, Shannon L. Fowler, Simon D. Goldsworthy, Robert Harcourt, Joseph I. Hoffman, Luis Hückstädt, Jana Jeglinski, Elizabeth A. McHuron, Clive R. McMahon, Sharon R. Melin, Diego Páez-Rosas, Svenja Stoehr, Fritz Trillmich, Stella Villegas-Amtmann, Rachel R. Holser.

Table S1. *Output of the linear mixed-effects model for post-dive duration (PDD, seconds). Predictors: maximum depth (meters) and dive duration (seconds), both standardized (mean = 0, SD = 1). Model: PDD ~ z(depth) * z(duration) + (1 + z(depth) || species) + (1 + z(depth) || individual).*

**Fixed effects**				
Predictors	*Estimates (s)*	*Std. Error (s)*	*R value*	*95% CI (s)*
Intercept	111.336	4.657	23.906	102.208 – 120.465
z(Max Depth)	24.858	5.062	4.911	14.937 – 34.779
z(Dive Duration)	-4.520	0.206	-21.993	-4.922 – -4.117
z(Max depth) x z(Dive duration)	13.216	0.111	119.056	12.998 – 13.433

**Random effects**			
[illegible]			
Group	*Effect*	*Variance (s2)*	*Std. Dev. (s)*
Species	Intercept	97.77	9.888
Species	z(Max depth) slope	116.72	10.804
Individual	Intercept	545.16	23.349
Individual	z(Max depth) slope	494.35	22.234
Residual	—	6401.36	80.008

Metric	*Value*
Observations	801,086
Individuals	374
Species	5
AIC	9,297,444
Marginal R2	0.210
Residual df	801,077
